# Effects of oral administration of lavender (*Lavandula angustifolia*) essential oil on growth performance, serum biochemistry and muscle fatty acid profile in Kangal Akkaraman lambs

**DOI:** 10.5194/aab-69-595-2026

**Published:** 2026-10-22

**Authors:** Abdullah Özbilgin, Halil İbrahim Toprak, Ömer Kayir

**Affiliations:** 1 Department of Animal Nutrition and Nutritional Diseases, Faculty of Veterinary Medicine, Sivas Cumhuriyet University, Sivas, Türkiye; 2 Ministry of Agriculture and Forestry of the Republic of Turkey, Samsun Havza District Agricultural Directorate, Samsun, Türkiye; 3 Hitit University Scientific and Technical Application and Research Center, Çorum, Türkiye

## Abstract

This study aimed to determine the effects of the direct oral administration of lavender (*Lavandula angustifolia*) essential oil on the growth performance, blood serum biochemistry and muscle fatty acid profile of lambs. For this purpose, an experiment was designed where pure lavender oil was administered individually to each animal via a syringe (excluding the control group). The study was conducted over 63 d , including a 7 d acclimatisation period and a 56 d experimental period, utilising 36 male Kangal Akkaraman lambs (12 weeks old, with an average initial body weight of 27.26 ± 3.06 kg ). The lambs were randomly assigned to three experimental groups (12 lambs per group) and fed the same basal diet ad libitum: Control (0 mg lavender oil per animal per day), Lav1000 (1000 mg lavender oil per animal per day) and Lav2000 (2000 mg lavender oil per animal per day). No statistically significant differences were observed among the groups regarding initial and final body weights, daily live weight gain, feed intake and the feed conversion ratio ( p > 0.05). Among the serum biochemical parameters, a statistically significant difference was detected exclusively in amylase and alkaline phosphatase (ALP) levels ( p < 0.05). In the fatty acid composition analysis, statistically significant differences were observed in cis-10-heptadecenoic acid, cis-11-eicosenoic acid and arachidic acid concentrations in the lavender-supplemented groups compared to the control group ( p < 0.05). In conclusion, determining the optimal dosage and administration method of lavender oil can provide critical insights for future research aimed at enhancing performance parameters, rumen fermentation, animal health and product quality in small ruminants.

## 1 Introduction

Lavender is a prominent member of the Lamiaceae family, deriving its name from the Latin verbs *lavo* and *lavare,* which mean “to wash” or “to clean”. Widely distributed across the Mediterranean region, this species is extensively cultivated in Italy, Spain and Türkiye. According to Dioscorides' historical publication *De Materia Medica*, lavender has long been utilised for medicinal purposes due to its therapeutic properties (Dioscorides, 1549). The Romans used lavender as a bath benefit, and it became one of the most valuable essential oil plants during the Middle Ages, utilised in perfumes, soaps, laxatives and food preparations. Lavender oil contains various monoterpenes as major bioactive components – such as linalool, linalyl acetate, lavandulol, geraniol, bornyl acetate, borneol, terpineol and lavandulyl acetate – which exhibit distinct antibacterial and antifungal activities depending on the chemical composition (Glinka and Glinka, 2008).

*Lavandula angustifolia* Mill. is recognised as an important aromatic and medicinal plant in traditional and alternative medicine, historically used to treat various gastrointestinal, rheumatic and neurological disorders (Hajhashemi et al., 2003). *Lavandula angustifolia* essential oil (EO) has been reported to possess anti-nociceptive, immunomodulatory and anti-inflammatory properties (Peana et al., 2002). Notably, historical scholars like Ibn Sina and Abu Bakr al-Razi recommended lavender for treating epilepsy and migraine attacks, and modern studies also support its analgesic and anti-tumour potential (Gorji, 2003). The antimicrobial efficacy of lavender oil is primarily attributed to high concentrations of linalool and linalyl acetate (Glinka and Glinka, 2008). Although commonly administered orally, lavender is also utilised via inhalation (Sasannejad et al., 2012), massage oils and baths (Xu et al., 2008). Recently, the incorporation of essential oils into animal nutrition has gained traction; the oils are reported to enhance dietary palatability, increase feed intake, regulate digestive functions and modulate gastrointestinal microflora. Consequently, these effects can improve the growth performance and feed conversion ratio (FCR) – an outcome of critical economic importance given rising feed costs (Adaszyńska-Skwirzyńska and Szczerbińska, 2019; Özbilgin and Kara, 2023; Özbilgin et al., 2023). However, conflicting studies indicate that using essential oils as growth promoters does not consistently improve performance parameters and may occasionally yield adverse effects (Kırkpınar et al., 2011; Saleh et al., 2014; Zeng et al., 2015).

Lavender oil, obtained from the flowering tops of the lavender plant, primarily contains volatile monoterpenes and sesquiterpenes such as linalool and linalyl acetate (Renaud et al., 2001; Adaszyńska-Skwirzyńska and Szczerbińska, 2018). Previous studies have mainly evaluated lavender supplementation through feed or drinking water, whereas information regarding direct oral administration and its effects on serum biochemistry and muscle fatty acid composition in lambs remains scarce. Recent studies have primarily focused on administering lavender essential oil via drinking water (Adaszyńska-Skwirzyńska and Szczerbińska, 2019) or incorporating it into diets at low concentrations (Küçükyılmaz et al., 2017). To ensure precise individual dosage control and to avoid feed palatability issues, the current study was designed to administer pure lavender oil orally to each animal (excluding the control group) via direct syringe injection. This study aims to comprehensively assess the positive effects of lavender essential oil on growth performance, blood serum biochemical profiles and fatty acid compositions. Furthermore, it is hypothesised that determining the correct therapeutic dosage will offer a viable alternative to conventional growth promoters without contributing to the global threat of antimicrobial resistance.

## 2 Method

All procedures involving animals were carried out with the approval of the Sivas Cumhuriyet University Local Ethics Committee for Animal Experiments (approval no. 608, year: 2023). A total of 21 d prior to the initiation of the study, all lambs received oral antiparasitic tablets for internal and external parasites, were vaccinated against enterotoxemia, and were administered vitamin and mineral injections. The study lasted for a total of 63 d , consisting of a 7 d acclimatisation period and a 56 d experimental period, and was conducted on 36 male Kangal Akkaraman lambs (12 weeks old, with an average initial body weight of 27.26 ± 3.06 kg ). The lambs were assigned to three homogeneous groups ( n=12 ) using a randomised complete block design based on initial body weight, ensuring no baseline statistical differences existed among groups ( P>0.05 ). Each lamb was housed in an individual pen (1.2 m × 1.5 m ) equipped with individual feed troughs and automatic drinkers to allow the precise daily measurement of individual feed intake and feed conversion ratio (experimental unit = individual animal). All groups were fed the same basal diet ad libitum. The three groups were designated as follows: Control (0 mg lavender oil per animal per day), Lav1000 (1000 mg lavender oil per animal per day) and Lav2000 (2000 mg lavender oil per animal per day). The selected daily doses (1000 and 2000 mg ) were determined based on previous literature on essential oil supplementation in small ruminants to evaluate dose-dependent responses within a safe threshold. Commercial lavender (*Lavandula angustifolia* Mill. subsp. *angustifolia*) essential oil was used in this study, and its chemical composition is reported in Table 1. Raw materials and nutritional content of lamb-growing feed and alfalfa hay formulated according to NRC (1985) standards are presented in Tables 2 and 3. The specified daily doses were administered orally as a single daily dose at 08:30–10:00 local time (prior to morning feeding) using a graduated syringe by the same trained operator throughout the experimental period to ensure standardisation and minimise handling stress.

**Table 1:** Chemical analysis of lavender oil.

Reaction time	Compound	%
7.11	Trans-sabinene hydrate	0.17
10.28	O-cymene	0.59
10.53	Eucalyptol	0.59
10.84	2(3H)-Furanone, 5-ethenyldihydro-5-methyl	0.63
12.26	Linalool oxide	8.03
12.92	Trans-linalool oxide	6.93
13.55	Linalool	19.52
13.92	1-Octen-3-yl-acetate	2.41
15.18	Camphor	0.37
16.32	Epoxylinalol	1.76
16.69	Terpinen-4-ol	2.76
18.70	6-Methyl-cyclodec-5-enol	0.60
20.32	Linalyl acetate	30.60
20.95	Farnesene	0.30
21.37	Bornyl acetate	0.33
21.67	Lavandulol	5.63
23.79	Hotrienol	3.73
24.19	2,6-Octadiene-1,8-diol, 2,6-dimethyl-	4.55
24.56	2,6-Dimethyl-1,7-octadiene-3,6-diol	0.76
25.04	Cyclopentanecarboxaldehyde	0.26
25.22	Berbenol	1.07
31.49	Caryophyllene oxide	8.43

**Table 2:** Lamb grower feed raw materials and nutrient concentrations.

Raw materials	%
Corn	42.62
Soybean meal	18.82
Wheat milling by-product (bonkalit)	10.00
Corn bran	8.70
Wheat bran, rough	8.27
Molasses	10.00
Marble dust	1.08
Vitamin-mineral premix	0.25
Salt	0.25
Analysis	%
DM^∗^	88.35
Crude protein^∗^	15.98
Crude cellulose^∗^	4.08
Crude ash^∗^	4.82
Ether extract^∗^	3.06
NDF^∗^	17.00
ADF^∗^	7.00
ME^∗^, $\mathrm{mcal}\;{\mathrm{kg}}^{-1}$	2.75
Starch^∗^	36.00
TDN^∗^	72.00
Ca ^∗^	0.60
P ^∗^	0.40
Na ^∗^	0.23

^∗^ DM: dry matter, NDF: nötral detergan fibre, ADF: acid detergan fibre, ME, $\mathrm{mcal}\;{\mathrm{kg}}^{-1}$ : metabolic energy megacalories/kilogram, TDN: total digestible nutrients, Ca : calcium, P : phosphorus, Na : sodium.

**Table 3:** Nutrient concentrations of alfalfa hay used in the feed.

Analysis	%
DM^∗^	97.46
Crude protein	15.76
Ether extract	1.93
Crude ash	10.40
ME, $\mathrm{mcal}\;{\mathrm{kg}}^{-1}$	1.86
NFC^∗^	24.62
Starch	5.60
NDF^∗^	44.76
ADF^∗^	38.17
Lignin	8.61
NDICP^∗^	1.06
ADICP^∗^	0.68
N fraction A, % HP	43.73
N fraction B, % HP	52.07
N fraction C, % HP	4.20
TDN^∗^	52.88

^∗^ DM: dry matter, NFC: non-cellulose carbohydrates, ME, $\mathrm{mcal}\;{\mathrm{kg}}^{-1}$ : metabolic energy (megacalories per kilogram), NDF: neutral detergent fibre, ADF: acid detergent fibre, NDICP: crude protein insoluble in neutral detergent, ADICP: crude protein insoluble in acid detergent, N fraction A, % HP: fraction A % crude protein, N fraction B, % HP: fraction B % crude protein, N fraction C, % HP: fraction C % crude protein, TDN: total digestible nutrients.

### 2.1 Gas chromatography-mass spectrometry (GC-MS) analysis of lavender oil

Gas chromatography (GC) analyses were performed using a Perkin-Elmer Clarus 500 Series system operating in split mode (split ratio 25:1 ), equipped with a flame ionisation detector (FID) and a TG-5MS apolar capillary column (30 m × 0.25 mm i.d., 0.25 µm film thickness) coupled to a mass spectrometer. The injector temperature was maintained at 250 °C . Helium was utilised as the carrier gas at a constant flow rate of 1.0 $\mathrm{mL}\;\min^{-1}$ . The oven temperature programme for the TG-5MS column was initiated at 50 °C , increased to 120 °C at a rate of 3 ${}^{\circ}C\;\min^{-1}$ , further increased to 220 °C at 3 ${}^{\circ}C\;\min^{-1}$ (held for 0.67 min^−1^) and finally raised to 250 °C at 5 ${}^{\circ}C\;\min^{-1}$ , where it was held for 5.0 min . For the mass spectrometer, the transfer line temperature was set to 250 °C , and the ionisation energy was maintained at 70 eV . Volatile compounds were identified by comparing their mass spectra with the WILEY and NIST libraries using relative peak areas.

### 2.2 Fattening performance parameters

The experimental period lasted for 9 weeks. Following the initial body weight measurement (day 0), a second weighing was performed on day 7 to conclude the acclimatisation period. Thereafter, the lambs were weighed individually every 14 d . Total live weight gain (LWG) was calculated by subtracting the initial body weight from the final body weight. Daily feed intake (FI) was determined by subtracting the refused feed from the total feed offered daily on a pen basis. The feed conversion ratio (FCR) was calculated as the ratio of daily feed intake to daily live weight gain.

### 2.3 Serum biochemical parameters

At the conclusion of the study (day 56), blood samples were collected from the left jugular vein of all lambs under sterile conditions following shaving and local disinfection of the neck area. Blood sampling was performed at 08:00 prior to the morning feeding following an overnight 12 h fasting period. Blood was drawn into 10 mL plain vacuum tubes containing clot activator and gel separator (BD Vacutainer^®^). The samples were allowed to clot in an upright position at room temperature (22–24 °C ) for 30 min . Blood serum was separated via centrifugation at 3000 × g for 15 min at 4 °C . Following centrifugation, serum was immediately visually inspected for haemolysis and lipemia; any haemolysed or severely lipemic samples were excluded from the analysis. Clear serum supernatant was aliquoted into multiple 1.5 mL microcentrifuge tubes to avoid repeated freeze–thaw cycles and stored at - 80 °C for a maximum duration of 2 months prior to analysis. Serum biochemical parameters (including glucose, total protein, albumin, blood urea nitrogen, creatinine, alanine aminotransferase (ALT), aspartate aminotransferase (AST), total cholesterol and triglycerides) were determined using commercial diagnostic reagent kits (Mindray Medical International Ltd.) according to the manufacturer's protocols on an automated analyser (Mindray BS200).

### 2.4 Fatty acid composition analysis

At the end of the study, six lambs randomly selected from each group were slaughtered under aseptic conditions. Post-slaughter, tissue samples were obtained from the *Musculus longissimus dorsi* of each carcass. Following homogenisation of the muscle tissue, total lipids were extracted using a chloroform/methanol solution. The fatty acid composition of the extracted lipid samples was determined via gas chromatography-flame ionisation detection (GC-FID) after conversion to fatty acid methyl esters (FAMEs). The extracted oils were saponified following standard IUPAC procedures. Subsequently, fatty acids were esterified using 10 % ( v/v ) methanolic boron trifluoride ( ${\mathrm{BF}}_{3}\text{-}\mathrm{MeOH}$ ) as the methylation reagent. The methylation of total lipids to obtain FAMEs was performed in accordance with official AOAC methods (Kisa et al., 2020; Yeşilayer and Genç, 2013).

Chromatographic analysis was carried out using a Thermo Scientific Trace 1310 GC system equipped with an FID and operated in split mode (split ratio 50:1 ). The system was fitted with an RTX-2330 apolar capillary column (30 m × 0.25 mm i.d., 0.2 µm film thickness; Restek). Helium served as the carrier gas at a flow rate of 1.0 $\mathrm{mL}\;\min^{-1}$ . The injector and detector temperatures were maintained at 250 °C and 260 °C , respectively. The oven temperature programme was configured as follows: initially held at 70 °C , increased to 120 °C at a rate of 4 ${}^{\circ}C\;\min^{-1}$ , then immediately increased to 180 °C at 2 ${}^{\circ}C\;\min^{-1}$ and held for 3 min . Subsequently, the temperature was raised to 200 °C at 4 ${}^{\circ}C\;\min^{-1}$ (held for 3 min ) and finally increased to 230 °C at 7 ${}^{\circ}C\;\min^{-1}$ with a final hold time of 5.21 min . Fatty acid peaks were identified by comparing their retention times with an authentic standard mixture (Supelco 37 Component FAME Mix). The relative concentration of individual fatty acids was expressed as a percentage of the total identified fatty acids based on the integrated peak areas.

### 2.5 Statistical analysis

Arithmetic means were used as descriptive statistics for the groups, and the standard error of the mean (SEM) was applied as a measure of distribution. The normality of the data distribution was assessed using the Kolmogorov–Smirnov test. One-way analysis of variance (ANOVA) was performed to determine the statistical significance of observed differences among the groups, and Duncan's post-hoc test was utilised for multiple comparisons. Statistical significance was declared at p<0.05 . All statistical analyses were executed using the IBM SPSS Statistics 20 software package (SPSS, 2011).

## 3 Result and discussion

No statistically significant differences were observed among the experimental groups regarding initial and final live weights, daily live weight gain, feed intake and the feed conversion ratio ( p > 0.05; Table 4).

**Table 4:** Effect of lavender oil on performance parameters in lambs.

Parameters	Control^a^	LAV1000^a^	LAV2000^a^	$p^{b}$
Live weight at the start of the experiment, kg	27 778 ± 1577	27 050 ± 0.833	26 090 ± 0.624	0.830
Weight at the end of the experiment, kg	35 300 ± 1191	35 410 ± 1212	34 680 ± 0.814	0.425
Daily live weight gain, g	149 745 ± 13 955	167 857 ± 10 841	142 857 ± 9109	0.362
Feed intake (roughage/concentrate), g	1 430 860 ± 14 995	1 419 477 ± 10 428	1 425 403 ± 39 825	0.119
Feed conservation ratio	10 072 ± 0.932	9585 ± 0.775	11 032 ± 1094	0.927

^a^ Mean ± standard error values were used for all groups. ^b^ p : statistical difference between experimental groups was determined to be significant at p<0.05 .

**Table 5:** Effect of lavender oil on serum biochemistry parameters in fattening lambs.

	Control		LAV1000^c^		LAV2000^c^		
	Mean^d^	SE^d^	Mean ^d^	SE^d^	Mean ^d^	SE^d^	p
Creatine, $\mathrm{mg}\;{\mathrm{dL}}^{-1}$	0.641	0.028	0.643	0.024	0.639	0.023	0.811
Amylase, U/L	12 250^a,b^	2097	20 600^a^	2731	7.600^b^	1435	0.004^c^
ALT, U/L^e^	20 714	2358	19 000	1195	26 143	2824	0.059
AST, U/L^e^	112 833	7259	114 400	11 285	121 667	4944	0.546
GGT, U/L^e^	60 143	4217	56 600	8698	64 714	3926	0.705
ALP, U/L^e^	332 100^a^	35 958	139 820^b^	10 933	276 600^a^	23 258	0.010^c^
CK, U/L^e^	562 667	94 626	786 250	77 245	728 833	70 129	0.914
TG, $\mathrm{mg}\;{\mathrm{dL}}^{-1}$ ^e^	18 900	0.713	13 743	1244	13 271	1175	0.158
Total cholesterol, $\mathrm{mg}\;{\mathrm{dL}}^{-1}$	55 643	1375	51 550	2707	53 257	3543	0.318
HDL, $\mathrm{mg}\;{\mathrm{dL}}^{-1}$ ^e^	19 767	1268	18 886	1680	20 671	1758	0.727
LDL, $\mathrm{mg}\;{\mathrm{dL}}^{-1}$ ^e^	40 167	1922	36 286	1643	35 143	2087	0.695
Total bilirubin, $\mathrm{mg}\;{\mathrm{dL}}^{-1}$	0.174	0.017	0.222	0.019	0.197	0.017	0.962
BUN, $\mathrm{mg}\;{\mathrm{dL}}^{-1}$ ^e^	23 286	1426	20 714	1358	22 857	2365	0.332
Total protein, $g\;{\mathrm{dL}}^{-1}$	7141	0.290	7251	0.349	7593	0.126	0.276
Albumin, $g\;{\mathrm{dL}}^{-1}$	2734	0.082	2770	0.067	2919	0.063	0.797
Globulin, $g\;{\mathrm{dL}}^{-1}$	4386	0.303	4486	0.345	4671	0.102	0.382
Glucose, $\mathrm{mg}\;{\mathrm{dL}}^{-1}$	27 667	5548	19 400	1691	19 333	0.955	0.070
Mg , $\mathrm{mg}\;{\mathrm{dL}}^{-1}$ ^e^	2987	0.223	3071	0.0788	3170	0.156	0.720
Ca , $\mathrm{mg}\;{\mathrm{dL}}^{-1}$ ^e^	11 832	0.300	11 993	0.337	12 346	0.256	0.694
P , $\mathrm{mg}\;{\mathrm{dL}}^{-1}$ ^e^	12 278	0.500	10 917	0.502	10 403	0.516	0.896

^a,b^ Differences between means indicated by different letters in the same row are significant ( P<0.05 ). ^c^ LAV1000: lavender 1000 mg , LAV2000: 2000 mg . ^d^ Mean: average, SE: standard error. ^e^ BUN: blood urea nitrogen, ALT: alanine aminotransferase, AST: aspartate aminotransferase, GGT: gamma-glutamyl transferase, CK: creatine kinase, TG: triglycerides, HDL: high-density lipoprotein, LDL: low-density lipoprotein, Mg : magnesium, Ca : calcium, P : phosphorus.

Regarding serum biochemical parameters, statistically significant differences among the treatment groups were detected in serum amylase and alkaline phosphatase (ALP) levels ( P<0.05 ; Table 5). In contrast, no statistically significant treatment effects were observed for creatinine, urea, blood urea nitrogen (BUN), total cholesterol, alanine aminotransferase (ALT), aspartate aminotransferase (AST), gamma-glutamyl transferase (GGT), creatine kinase (CK), total bilirubin, phosphorus, high-density lipoprotein (HDL), low-density lipoprotein (LDL), triglycerides (TG), albumin, total protein, globulin, albumin/globulin (A/G) ratio, glucose, magnesium or calcium ( P>0.05 ).

**Table 6:** The effect of lavender oil on individual and total fatty acid profile in lamb meat.

	CONTROL	LAV1000	LAV2000	P
C6:0 (caproic acid)	0.043 ± 0.016	0.079 ± 0.024	0.077 ± 0.028	0.541
C8:0 (caprylic acid)	0.465 ± 0.152	0.777 ± 0.309	0.660 ± 0.160	0.620
C12:0 (lauric acid)	0.168 ± 0.015	0.146 ± 0.013	0.125 ± 0.032	0.468
C14:0 (myristic acid)	0.166 ± 0.009	0.260 ± 0.019	0.192 ± 0.032	0.121
C14:1 (myristoleic acid)	2999 ± 0.113	2640 ± 0.297	2517 ± 0.234	0.413
C15:0 (pentadecanoic acid)	0.089 ± 0.014	0.090 ± 0.000	0.061 ± 0.015	0.289
C16:0 (palmitic acid)	0.456 ± 0.036	0.333 ± 0.032	0.319 ± 0.026	0.096
C16:1 (palmitoleic acid)	29 948 ± 1148	31 079 ± 0.082	30 692 ± 0.921	0.672
C17:0 (heptadecanoic acid)	1143 ± 0.229	1038 ± 0.160	0.798 ± 0.073	0.432
C17:1 (cis-10-heptadecenoic acid)	1487 ± 0.049^a^	0.879 ± 0.024^b^	1020 ± 0.034^b^	0.003^c^
C18:0 (stearic acid)	0.639 ± 0.018	0.527 ± 0.049	0.444 ± 0.051	0.103
C18:1n9c (oleic acid)	18 352 ± 0.191	18 938 ± 0.774	21 567 ± 1147	0.125
C18:2n6c (linoleic acid)	35 402 ± 0.866	27 767 ± 2370	29 957 ± 2326	0.145
C20:0 (arachidic acid)	5744 ± 0.289	9993 ± 0.992	7864 ± 0.329	0.039^c^
C18:3n3 ( α -linolenic acid)	0.102 ± 0.013	0.081 ± 0.081	0.153 ± 0.005	0.604
C20:1n9 (cis-11-eicosenoic acid)	0.496 ± 0.003^b^	0.750 ± 0.041^a^	0.713 ± 0.005^a^	0.009^c^
C20:2(cis-11,14-eicosadienoic: acid)	0.658 ± 0.047	0.552 ± 0.047	0.591 ± 0.037	0.357
C20:3n3 (cis-11,14,17-eicosatrienoic acid)	0.128 ± 0.009	0.222 ± 0.012	0.038 ± 0.037	0.126
C20:3n6 (cis-8,11,14-eicosatrienoic acid)	0.107 ± 0.029	0.181 ± 0.053	0.143 ± 0.036	0.516
C22:2 (cis-13,16-docosadienoic acid)	0.924 ± 0.153	1834 ± 0.001	1485 ± 0.286	0.250
C24:0 (lignoceric acid)	0.141 ± 0.003	0.372 ± 0.080	0.224 ± 0.037	0.105
Saturated fatty acid (SFA) %	54 370 ± 1402	55 580 ± 0.594	57 483 ± 1846	0.394
Unsaturated fatty acid (UFA) %	45 629 ± 1403	44 417 ± 0.588	42 515 ± 1849	0.394
Saturated/unsaturated fatty acid orani (SFA/UFA) %	1194 ± 0.067	1252 ± 0.029	1357 ± 0.102	0.396
Mono unsaturated fatty acid (MUFA) %	38 058 ± 0.967	30 123 ± 2677	31 939 ± 2591	0.164
Poli unsaturated fatty acid (PUFA) %	7571 ± 0.436	14 294 ± 2088	10 577 ± 0.742	0.079

^a,b^ Differences between means indicated by different letters in the same row are significant ( P < 0.05). ^c^ Mean ± standard error values were used for all groups. ^d^ p : statistical difference between experimental groups was determined to be significant at p<0.05 .

In the fatty acid composition analysis, statistically significant differences were determined among the experimental groups in terms of cis-10-heptadecenoic acid, cis-11-eicosenoic acid and arachidic acid concentrations ( p < 0.05; Table 6).

The active compounds in phytogenic oils are suggested to modulate gut microbial flora, alter ruminal fermentation patterns and stimulate the secretion of endogenous digestive enzymes, thereby potentially enhancing growth performance in farm animals (Brenes and Roura, 2010; Smeti et al., 2015). In small ruminants, essential oil supplementation has generally been reported to influence growth performance and nutrient digestibility by modulating ruminal volatile fatty acid production and inhibiting hyper-ammonia-producing bacteria (Parvar et al., 2018). Specifically, the dietary inclusion of Lamiaceae family plant extracts, such as thyme and rosemary, demonstrated positive effects on daily weight gain and feed conversion ratio in fattening lambs (Bampidis et al., 2005; Giannenas et al., 2018). However, responses to essential oils in ruminants remain inconsistent; several authors observed no significant improvements in performance parameters when feeding plant extracts to small ruminants (Botsoglou et al., 2002; Reisinger et al., 2011), which aligns with our present findings. In the present study, no statistically significant differences were observed among groups for final live weight, average daily gain or feed conversion ratio ( p>0.05 ). Nevertheless, a numerical increase in final live weight and average daily gain was noted in the Lav1000 group compared to the control group, suggesting a potential positive trend at moderate doses. Owing to the limited body of literature evaluating pure lavender essential oil in small ruminants, indirect evidence from monogastric models (e.g. poultry) suggests that lavender oil active compounds, such as linalool, may stimulate digestive enzyme secretion and improve feed efficiency (Nasiri-Moghaddam et al., 2012; Adaszyńska-Skwirzyńska and Szczerbińska, 2018). However, because essential oils undergo extensive bio-transformation by ruminal microbiota in lambs, direct extrapolation from monogastrics should be interpreted with caution. The substantial variability in the efficacy of phytogenic feed additives on lamb growth performance across different studies may stem from variations in animal genetics, basal diet composition, ruminal microbial adaptation, plant chemotypes and administration methods.

Regarding serum biochemical profiles, the oral administration of lavender essential oil did not result in statistically significant changes in serum triglyceride ( p=0.158 ) or LDL cholesterol ( p=0.695 ) concentrations among the treatment groups. Although mean values for triglycerides and LDL cholesterol were numerically lower in the Lav1000 and Lav2000 groups compared to the control group, these numerical variations must be interpreted with caution as they do not represent a definitive lipid-lowering effect. In literature evaluating phytogenic additives, the absence of significant alterations in blood biochemical markers is frequently reported (Najafi and Torki, 2010; Khattak et al., 2014; Kim et al., 2016). For instance, Adaszyńska-Skwirzyńska and Szczerbińska (2019) similarly observed no significant modifications in circulating cholesterol or triglyceride levels following lavender oil supplementation. Hypothetically, active monoterpenes present in lavender essential oil – specifically linalool and linalyl acetate (as characterised in Table 1) – have been speculated in physiological literature to modulate hepatic cholesterol biosynthesis by inhibiting β -hydroxy β -methylglutaryl-CoA (HMG-CoA) reductase activity. However, because hepatic enzyme activities were not directly evaluated in the present study, this mechanism remains speculative. Furthermore, while essential oil supplementation in ruminants can indirectly influence systemic energy metabolism by altering ruminal propionate production and downstream metabolites such as non-esterified fatty acids (NEFA) and β -hydroxybutyrate (BHBA) (Soltan et al., 2018; Orzuna-Orzuna et al., 2022), ruminal fermentation parameters were not measured in this trial. Therefore, whether the observed numerical trends in serum lipids are related to ruminal microbial shifts or hepatic lipid metabolism warrants further investigation in studies incorporating ruminal VFA and liver enzyme assays. This implies that essential oils can improve energy balance in small ruminants. Although information on the precise mechanisms of essential oils on lipid metabolism in small ruminants remains limited, specific terpenoids have been shown in murine models to inhibit hepatic cholesterol biosynthesis and downregulate genes involved in fatty acid synthesis, such as *Fas*, *Scd1* and *Acc1* (Jun et al., 2015). Similar biochemical pathways triggered by lavender oil components may explain the lower serum LDL and triglyceride levels observed in this study. Furthermore, lavender administration had no significant effect on serum albumin, globulin or the albumin : globulin ratio. This aligns with previous reports stating that supplemental essential oils do not alter these protein fractions, indicating no adverse effects on protein catabolism or overall nutritional status in small ruminants (Orzuna-Orzuna et al., 2022).

In the present study, blood serum urea nitrogen (BUN) concentrations were not significantly affected by lavender essential oil administration ( P>0.05 , Table 5), remaining within standard physiological ranges for growing lambs. Although previous meta-analyses and literature on essential oil supplementation in small ruminants have occasionally reported reductions in BUN – hypothesised to stem from reduced ruminal deamination by hyper-ammonia-producing bacteria (Orzuna-Orzuna et al., 2022) – no such effect was observed under the conditions of our study. Furthermore, because ruminal fermentation characteristics (such as ruminal ${\mathrm{NH}}_{3}\text{-}N$ and short-chain fatty acid concentrations) were not evaluated in the present trial, any potential influence of lavender oil on ruminal nitrogen partitioning remains purely speculative. Similarly, although active monoterpenes in essential oils are widely recognised in literature for their antioxidant potential (Vasta and Luciano, 2011), systemic antioxidant markers were not quantified in this study and warrant direct evaluation in future investigations.

Numerous investigations have evaluated the effects of dietary essential oils on the muscle fatty acid profiles of ruminants. In ruminants, dietary unsaturated fatty acids undergo extensive microbial biohydrogenation by ruminal microorganisms (such as *Butyrivibrio fibrisolvens*), converting polyunsaturated fatty acids (PUFA) into saturated fatty acids (SFA) prior to intestinal absorption. Bioactive compounds present in essential oils can modulate this process by selectively influencing biohydrogenating bacteria, potentially altering the fatty acid composition reaching the post-ruminal tissues (Vasta and Luciano, 2011; Chikwanha et al., 2018). In the present study, the oral administration of lavender essential oil induced specific changes in long-chain fatty acid proportions in lamb muscle tissue. Compared to the control group, significant increases were detected in cis-11-eicosenoic acid ( C20:1c11 ) and arachidic acid ( C20:0 ) proportions in the lavender-supplemented groups ( P<0.05 ). Conversely, a statistically significant reduction was observed in cis-10-heptadecenoic acid ( C17:1c10 ) levels ( P<0.05 ). Rather than representing an overall improvement in the nutritional value of the meat, these shifts reflect specific alterations in minor long-chain saturated and monounsaturated fatty acid fractions. Notably, arachidic acid is a long-chain saturated fatty acid; thus, its accumulation should be described neutrally rather than as a favourable outcome for human health. These observed variations suggest that lavender oil active metabolites (primarily linalool and linalyl acetate) exerted a localised or minor influence on ruminal biohydrogenation kinetics or tissue fatty acid incorporation, although the broader nutritional and organoleptic implications of these minor shifts remain limited and require further research.

Overall, no detrimental shifts in individual or total fatty acid concentrations occurred as a result of ruminal biohydrogenation. This is consistent with studies reporting that lavender oil supplementation does not adversely alter the baseline fatty acid matrix of meat products. Despite the extensive breakdown of unsaturated fatty acids by rumen microorganisms, strategic dietary lipid or essential oil supplementation remains the most viable method to favourably manipulate the fatty acid profile of ruminant products (Alvarenga et al., 2015; Jaturasitha et al., 2016; Chikwanha et al., 2018).

## 4 Conclusions and outlook

In conclusion, the present study demonstrated that the oral administration of lavender essential oil at tested dose levels did not alter growth performance, blood serum biochemical parameters (including BUN, triglycerides and cholesterol levels) or overall health status in growing Kangal Akkaraman lambs. However, lavender essential oil supplementation exerted specific minor shifts in muscle fatty acid composition, significantly increasing cis-11-eicosenoic acid (C20:1c11) and arachidic acid (C20:0) proportions while decreasing cis-10-heptadecenoic acid (C17:1c10) levels. Given that these fatty acid modifications carry limited nutritional implications and ruminal fermentation parameters were not directly quantified, further comprehensive studies incorporating ruminal sampling, volatile fatty acid profiling and oxidative stress markers are warranted to elucidate the precise metabolic pathways of lavender essential oil in small ruminants. The present study demonstrates that the direct oral administration of lavender (*Lavandula angustifolia*) essential oil to male Kangal Akkaraman lambs at doses of 1000 and 2000 mg does not adversely affect the growth performance, feed intake or feed conversion ratio. The oral administration of lavender essential oil did not result in statistically significant alterations in serum triglyceride ( P=0.158 ) or LDL cholesterol ( P=0.695 ) concentrations among the treatment groups (Table 5). Although mean values for serum triglycerides and LDL cholesterol were numerically lower in the Lav1000 and Lav2000 groups compared to the control group, these non-significant numerical variations cannot be interpreted as evidence of an improved lipid metabolism or enhanced metabolic health. Furthermore, all measured serum lipid parameters across both control and treatment groups remained well within established physiological reference intervals for growing lambs (e.g. triglycerides: 0.15–0.50 $\mathrm{mmol}\;L^{-1}$ ; total cholesterol: 1.3–3.2 $\mathrm{mmol}\;L^{-1}$ ). Because baseline (pre-experimental) serum biochemical values were not recorded prior to treatment initiation, and post-treatment differences lacked statistical significance, these numerical shifts reflect normal biological variations rather than a definitive treatment-induced hypolipidemic effect. Future studies evaluating phytogenic feed additives in small ruminants should incorporate pre-trial baseline blood sampling and age-matched clinical reference ranges to better assess potential subtle shifts in systemic lipid homeostasis.

In conclusion, establishing precise alternative delivery methods and therapeutic dosages – such as individual oral administration – can maximise the beneficial systemic effects of essential oils on animal health and product quality. These findings suggest that lavender essential oil has the potential to serve as a safe, natural bio-additive in small ruminant production, offering a viable alternative to synthetic growth promoters without contributing to the global risk of antimicrobial resistance. Future long-term studies are recommended to further elucidate the underlying molecular mechanisms of lavender oil on ruminal fermentation and tissue-specific gene expressions.

## Data Availability

The datasets generated during and/or analysed during the current study are not publicly available due to university data restrictions but are available from the corresponding author on reasonable request.
